# An RNAi-Based Dimorphic Genetic Screen Identified the Double Bromodomain Protein BET-1 as a Sumo-Dependent Attenuator of RAS-Mediated Signalling

**DOI:** 10.1371/journal.pone.0083659

**Published:** 2013-12-10

**Authors:** Fiona Gee, Kate Fisher, Ulrike Klemstein, Gino B. Poulin

**Affiliations:** Faculty of Life Sciences, The University of Manchester, Manchester, Greater Manchester, United Kingdom; CRG, Spain

## Abstract

Attenuation of RAS/RAF/MAPK signalling is essential to prevent hyperactivation of this oncogenic pathway. In *C. elegans*, the sumoylation pathway and a combination of histone tail modifications regulate gene expression to attenuate the LET-60 (RAS) signalling pathway. We hypothesised that a number of chromatin regulators are likely to depend on sumoylation to attenuate the pathway. To reveal these, we designed an RNAi-based dimorphic genetic screen that selects candidates based on their ability to act as enhancers of a sumo mutant phenotype, such interactions would suggest that the candidates may be physically associated with sumoylation. We found 16 enhancers, one of which BET-1, is a conserved double bromodomain containing protein. We further characterised BET-1 and showed that it can physically associate with SMO-1 and UBC-9, and that it can be sumoylated *in vitro* within the second bromodomain at lysine 252. Previous work has shown that BET-1 can bind acetyl-lysines on histone tails to influence gene expression. In conclusion, our screening approach has identified BET-1 as a Sumo-dependent attenuator of LET-60-mediated signalling and our characterisation suggests that BET-1 can be sumoylated.

## Introduction

**Figure 1:**
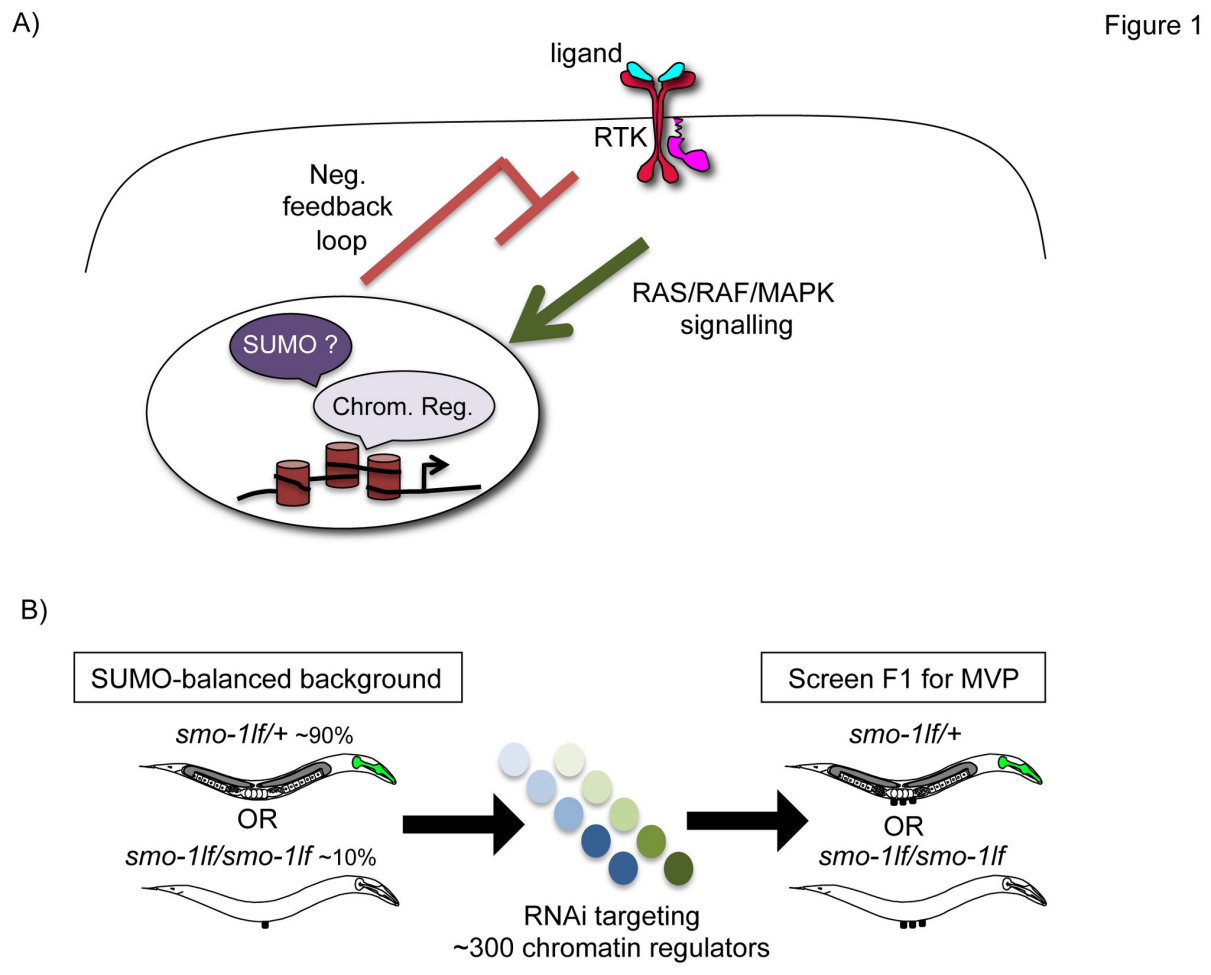
A targeted RNAi screen for SUMO interactors that attenuate LET-60-mediated signalling. A) Activation (green arrow) of LET-60-mediated signalling in the vulva by the LIN-3 (EGF), LET-23 (RTK), LET-60 (RAS), LIN-45 (RAF), and MPK-1 (MAPK) signalling cascade. Negative feedback loop is produced by chromatin regulators and the sumoylation pathway to attenuate LET-60-mediated signalling (in red). B) Diagram depicting the RNAi screening approach in a SUMO-balanced background using the MVP (multiple ventral protrusions) phenotype as readout for hyperactivation of the LET-60 signalling pathway during vulval development. The green pharynx indicates GFP balanced animals lacking one copy of *smo-1*, the non-green animals are *smo-1*_*lf*_ */smo-1*_*lf*_ homozygotes. The percentage indicates the approximate ratio of adult animals for each genotype observed. *smo-1*_*lf*_ */smo-1*_*lf*_ are 100% sterile presenting a *p*rotruding *v*u*l*va (PVL) and about 10% of these Pvl animals will display the *mu*lti*v*ulvae (MUV) phenotype. This was taken into account during the screen (see Materials and Methods).

It has been long established that the conserved RAS/RAF/MAPK signalling pathway can act as an oncogenic pathway in different types of cancer [1,2,3,4]. Normal cells possess the capacity to attenuate the activated RAS/RAF/MAPK signalling cascade, and a major contributor to attenuation is the induction of a transcriptional negative feedback loop [5,6,7]. In *C. elegans*, growth factors such as EGF or FGF mostly signal *via* activation of LET-60 (RAS) and recruitment of LIN-45 (RAF) to the plasma membrane [8]. This leads to a series of phosphorylation events that culminate in the transfer of MPK-1 (MAPK) to the nucleus. Here, MAPK phosphorylates an array of nuclear targets, including transcription factors and components of chromatin modifying complexes [8]. This impacts on gene expression and produces a negative feedback loop important to prevent hyperactivation of the RAS/RAF/MAPK signalling cascade (Figure 1A) [5,6,7]. 

Specific chromatin regulators, transcription factors, and the sumoylation pathway have been identified as attenuators of LET-60 (RAS)-mediated signalling in *C. elegans* [9,10,11,12,13]. During sumoylation a short polypeptide, SUMO (*S*mall Ubiquitin-like *MO*difier) is transferred onto lysines of specific substrates *via* a cascade of enzymatic reactions (E1, E2 and E3) akin to ubiquitination [14,15]. Sumoylation can control protein-protein interactions by producing a SUMO binding interface recognised by proteins containing a SUMO Interacting Motif (SIM) [16,17,18,19]. It has been shown in many species, including *C. elegans*, that sumoylation can affect gene expression through regulation of transcription. For example, the SUMO-modified form of the transcription factor LIN-1 (a *C. elegans* ETS transcription factor) is recognised by MEP-1, a component of the NuRD (*Nu*cleosome *R*emodelling Deacetylase) complex. This recruitment leads to repression of transcription [20]. Similarly, in *Drosophila*, sumoylated Sp3 can recruit NuRD to repress transcription [21]. Thus, a model by which sumoylation may attenuate LET-60 signalling is by first targeting proteins for direct modification followed by the recruitment of SIM-containing proteins. This sequence of events would regulate the expression of genes and lead to attenuation of the LET-60 signalling pathway.

Here we report on a screen to identify SUMO-dependent attenuators of LET-60 signalling and on the characterisation of a candidate, BET-1. BET-1 is a conserved double bromodomain protein recognising acteyl-lysines on histone tails [22]. Our results revealed that BET-1 requires sumoylation, most likely as a target, to attenuate the LET-60 signalling pathway. This is supported by results showing that BET-1 physically interacts with SMO-1 and UBC-9, and that BET-1 can be sumoylated *in vitro*. Moreover, we identified lysine 252 within the conserved second bromodomain as essential for sumoylation. We therefore suggest a speculative model by which BET-1’s ability to recognise acetyl-lysines is regulated by sumoylation. This effect would ensure the establishment of a transcriptional negative feedback loop necessary to attenuate LET-60 signalling. 

## Materials and Methods

### Strains and general maintenance

Strains were maintained at 20°C as described in [23], unless stated. The list of all strains used with their complete genotype is provided in Table S3. All experiments were performed with *bet-1*_*(gk425)*_ referred to as *bet-1*_*lf*_ unless stated. The *smo-1* allele (*ok359*) is a deletion producing a total knock out of the *smo-1* gene [9,20,24] and is referred to as *smo-1*_*lf*_.

### Identification of putative chromatin regulators

The RNAi screen targeted predicted chromatin regulators based on the presence of at least one domain found in known chromatin regulators. This search led to the identification of over 300 genes (Tables S1 and S2). The Pfam accession number for each domain of interest was used to search the WormBase database (release WS190), using the WormMart data mining tool (http://www.wormbase.org/biomart/martview). 

### RNAi experiments

RNAi screens of the 324 gene set were performed similarly to those described previously [9,25]. Briefly, individual cultures were used to inoculate three wells on a six-well plate, around ten synchronized *smo-1*_*lf*_ */hT2* L3-L4 stage worms were placed in the upper well for each bacterial strain and the plates maintained at 20 °C. After 48 h, five worms from the upper well were transferred to the lower well. The F_1_ progeny were scored for the MVP (multiple ventral protrusion) phenotype [13]. The MVP phenotype is the superficial manifestation of the *Mu*lti*V*ulvae (Muv) phenotype [26,27,28]. RNAi clones giving MVP in one or more *smo-1*_*lf*_ */hT2* animals (GFP positive) and/or two or more *smo-1*_*lf*_ */smo-1*_*lf*_ animals (GFP negative) were selected for further analysis. The complete loss of SMO-1 produces sterile escapers, some of which (~10%) display the Muv phenotype [9,20,24]; this was taken into account. RNAi clones were obtained from the Ahringer RNAi library [25] and the Vidal RNAi library [29]. All positive RNAi clones were verified by sequencing. All further RNAi experiments were performed as described above, with the exception that around ten worms were placed in the left hand well, and five worms were transferred to the middle well and then to the right hand well.

### General methods for nucleic acid manipulations

All plasmids were verified by sequencing. The full length *bet-1* ORF and various fragments of the *bet-1* ORF were PCR amplified from N2 cDNA and cloned into the Gateway system (Invitrogen). For yeast-two hybrid, *bet-1* bait inserts were cloned into pLexA-NLS using primer derived BamHI and PstI sites; the *smo-1* insert was cloned into pACT using primer derived BamHI sites.

### Western blot analysis

Whole worm protein extracts were prepared by harvesting synchronized L4 worms, washing the pellet in 1x PBS and boiling in 1x sample buffer containing 100 mM DTT, and sonicating. Antibodies against tubulin and Erk were used for normalization. Antibodies used were anti-phospho-p44/42 MAPK (Cell Signaling Technology, ♯9106), anti-p44/42 MAPK (Cell Signaling Technology, ♯4695) and mouse monoclonal anti-tubulin. Detection was performed using the Amersham ECL Plus Western Blotting Detection system (GE Healthcare) according to the manufacturer’s instructions.

### Yeast two hybrid assays

Yeast were transformed with a pLexA-NLS bait plasmid containing full length *bet-1* ORF or *bet-1* ORF fragments, and pACT prey plasmid containing mature *smo-1* or *ubc-9* ORF. pLexA-NLS TBX-2 and pACT UBC-9 were used as a positive control [30] (data not shown). Briefly, positive transformants were used to inoculate 5 ml CSM-Leu-Trp and grown for 24 hours at 30°C with shaking. OD_600_ readings of the saturated cultures were measured and used to adjust the concentration. Each yeast culture was transferred to an Eppendorf and the cells pelleted. Each pellet was then resuspended in 750 µl water. A total of 6 serial 1:5 dilutions were spotted from left to right with decreasing concentration on CSM-Leu-Trp and CSM-Leu-Trp-His plates and incubated at 30°C for 72 hours. 

### *In vitro* SUMO assay

The SUMO assay was performed as recommended by the manufacturer (ENZO Life Sciences). The proteins tested in the assay were MBP fusions and purified according to manufacturer’s recommendations. Briefly, the cell pellet was freeze-thawed, sonicated in lysis buffer and the lysate was applied to 100 μl of the appropriate beads (Amylose Resin, NEB). All purified proteins were checked by separation on a polyacrylamide gel followed by Coomassie Blue staining.

### The *egl-17*::*cfp* assay

In this assay, *egl-17::cfp* expression is detected in the daughter (.x) and grand daughter cells (.xx) of the VPCs (vulval precursor cells: P3.p to P8.p) displaying high levels of LET-60 (RAS) signalling. In wild type, only the descendants of P6.p express *egl-17::cfp*; the descendants of P3.p, P4.p, P5.p and P7.p, P8.p are *egl-17::cfp* negative due to low levels of LET-60 RAS signalling. This established assay was previously described in detail [13,31]. We did not score at the 3-cell stage or the 22-cell stage of vulval development, since the assay is not valid at these stages.

## Results

### An RNAi-based dimorphic genetic screen to identify SUMO interactors

Our main interest is to understand the role that chromatin regulators play in attenuation of LET-60-mediated signalling. Previous studies suggested that many chromatin regulators are SUMO-modified ([32,33,34] or able to recognise SUMO-modified targets through SIMs (SUMO Interacting Motifs) [21,35]. Further, the sumoylation pathway itself was shown to attenuate LET-60-mediated signalling [9,20,24]. We sought to identify the chromatin regulators that could be directly sumoylated, reasoning that the molecular function of these candidates may be regulated by sumoylation and that this regulatory activity would be important to achieve attenuation of the LET-60 signalling pathway. We designed a targeted RNAi screen that could enrich for SUMO-modified candidates. The screen was performed in a *sumo* knock out background maintained viable as a genetically and GFP balanced heterozygote (Figure 1A). This balanced strain gives rise to two types of progeny: GFP negative homozygotes (*smo-1*_*lf*_ */*_*lf*_; traces of maternally inherited SUMO) or GFP positive heterozygotes (*smo-1*_*lf*_ */+*; reduced SUMO levels) (Figure 1B). Following RNAi treatments applied to the balanced strain, we scored the progeny for a phenotype suggesting hyperactivation of the LET-60 signalling pathway, the *m*ultiple *v*entral *p*rotrusion phenotype (MVP) [13]. Importantly, we noted in which backgrounds (GFP negative or positive) the MVP phenotype was observed (Table 1). For convenience, we called the candidates producing the MVP phenotype in the GFP negative background (traces of SUMO) synthetic interactors and the candidates found in the GFP positive background (reduced SUMO levels) enhancers. 

**Table 1:** Candidates identified by an RNAi screen targeting predicted chromatin regulators in a SUMO-balanced background.

targeted genes	protein domain(s)	human homologs	Type of SUMO interactor	*s/s* or *s/*+
*athp-1*	Phd	*PHF12*	unknown	*s/s*
*bet-1*	Bromo	*BRD4*	unknown	*s/+*
*brd-1*	Brct	*BARD1*	unknown	*s/s*
*jmjd-5*	hat, jumonji	*JMJD5*	unknown	both
*dpff-1*	Phd	*DPF3*	unknown	*s/s*
*cec-9*	chromo	?	NA	*s/s*
*cdt-2*	WD40	*DTL*	unknown	*s/s*
*chd-3*	chromo, phd, snf2	*CHD3*	SUMO-binding	*s/s*
*cec-5*	chromo	?	NA	*s/s*
*jmjd1.1*	jumonji, phd	*JHDM1D*	unknown	*s/+*
*F47D12.9*	WD40	*DCAF4L2*	unknown	*s/s*
*gei-17*	ubiquitin	*PIAS1*	CORE	*s/s*
*gei-8*	sant myb	*NCOR1*	TARGET	*s/s*
*hda-2*	hdac	*HDAC1*	TARGET/SUMO-binding	both
*hda-6*	hdac	*HDAC10*	unknown	both
*hpl-2*	chromo	*HP1*	TARGET	both
*wdr-5.2*	WD40	*WDR5*	unknown	both
*lin-53*	WD40	*RbAp48/46*	SUMO-binding	both
*mes-2*	hmt	*EZH2*	TARGET	both
*mes-4*	hmt	*WHSC1*	unknown	both
*met-2*	hmt	*SETDB1*	SUMO-binding	*s/+*
*mys-1*	hat	*TIP60*	TARGET	both
*rbbp-5*	WD40	*RBBP5*	unknown	both
*set-1*	hmt	*SETD8*	unknown	both
*tag-250*	tudor	?	NA	*s/s*
*uba-2*	ubiquitin	*UBA2*	CORE	both
*wdr-5.1*	WD40	*WDR5*	unknown	*s/s*
*rcor-1*	sant myb	*RCOR3*	unknown	*s/+*

The type of SUMO interactor is based on what is known in other organisms. s/s indicates interaction observed in *smo-1* deleted homozygotes, s/+ heterozygotes, and both indicates observed in both backgrounds.

We found 28 SUMO interactors forming three groups: 12 synthetic candidates, 4 enhancers, and 12 that produce synthetic and enhancer interactions (Table 1). To our knowledge, none of these candidates have previously been shown to be associated with sumoylation, either as targets or as SUMO-binding proteins, in *C. elegans*. 25 of these candidates have a putative human homolog, and a number of these homologs have been shown to be sumoylated (*TIP60* [36], *EZH2* [37], *HDAC1* [38], *HP1* [39], and *NCOR1* [40]) or to act as SUMO-binding proteins (*SETDB1* [41], *CHD3* [41,42] and *RbAp48* [43]) (Table 1). We therefore conclude that this novel screening approach has identified both new SUMO substrates and SUMO-binding proteins in *C. elegans*.

### BET-1 is an attenuator of LET-60 (RAS) signalling

**Figure 2:**
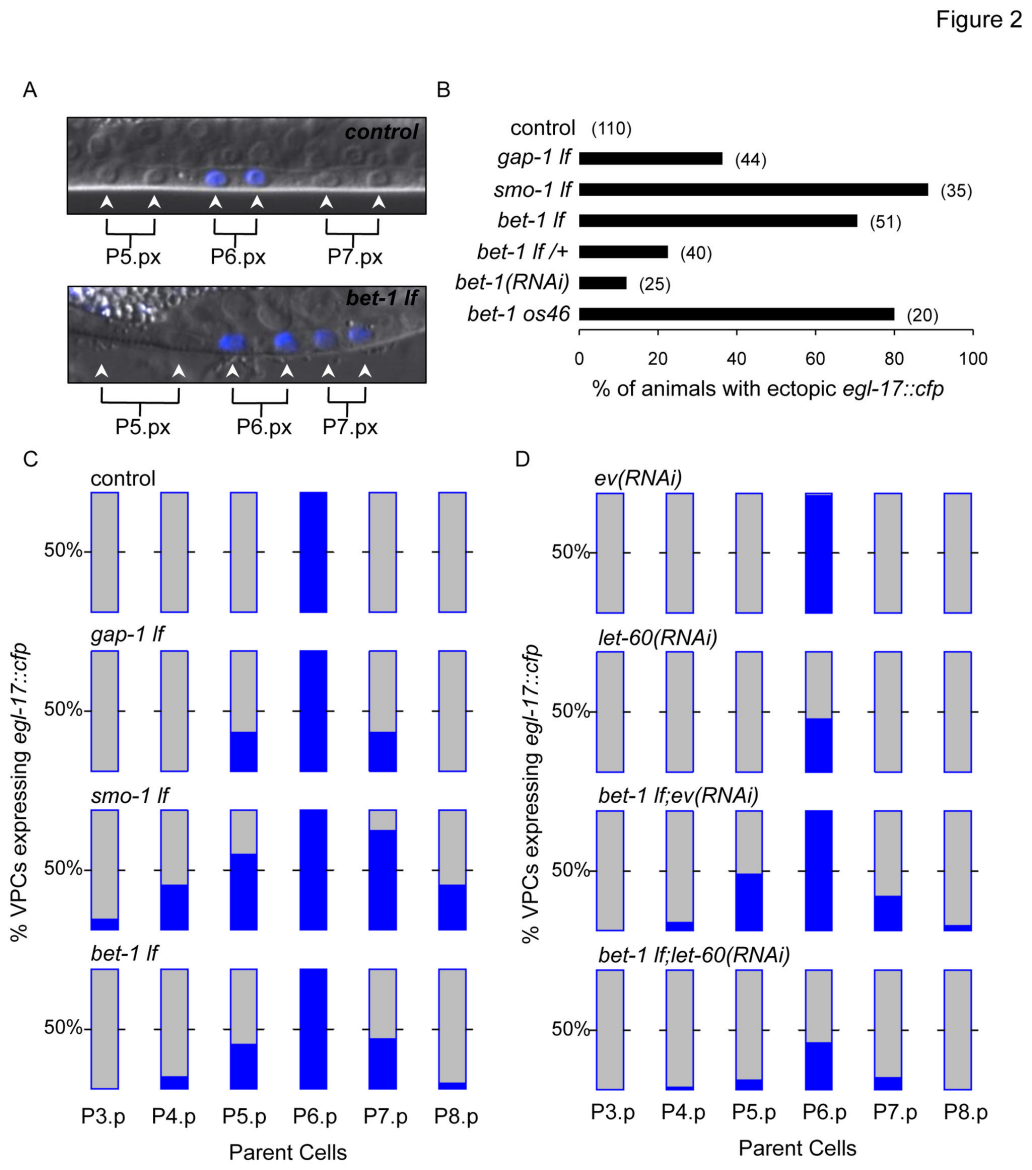
Analysis of *egl-17::cfp* expression. A) DIC and fluorescence photographs showing *egl-17::cfp* expression (in cyan blue) in wild type (N2) *versus* bet*-*1_*lf*_. Arrowheads indicate VPCs. B) Absence of *smo-1* or bet*-*1 causes *egl-17::cfp* ectopic expression. A gap-*1* (GTPase Activating Protein, a known negative modulator of the pathway [44]) loss of function mutant (gap-1_ga133_) is also shown. C and D) Detailed analysis of the VPCs expressing *egl-17::cfp*. Each bar represents the descendants of the indicated parental VPC with the 50% ectopic expression mark indicated. We analysed a minimum of 30 animals for each genotype. The genotype analysed is indicated in the top left hand corner.

The screen potentially revealed new attenuators of the LET-60 signalling pathway based on the manifestation of the MVP phenotype in a SUMO-impaired background, but the screen did not assess whether the candidates can attenuate LET-60 signalling in the wild type background. To assess the candidates’ capacity to attenuate LET-60 signalling, we used a reporter that can detect elevated LET-60 signalling, as observed by the depletion of known negative modulators such as GAP-1 [44]. GAP-1 is GTPase activating protein, increasing the exchange of GTP (active) for GDP (inactive). Thus, to complement and validate our screen results, we used this reporter system to identify the candidates that can prevent hyperactivation of LET-60 signalling on their own. Crucially, this assay is quantitative and provides cellular resolution, indicating the frequency at which vulval cells display hyperactivation of the LET-60 signalling pathway. The reporter is based on an adapted *egl-17*_*promoter*_ fused to *cfp* to produce a nuclear reporter system [31]. CFP is expressed only in Vulval Precursor Cells (VPCs) displaying high levels of LET-60 signalling (see Materials and Methods for details). Briefly, amongst the six VPCs (P3.p-P8.p) of a wild type animal, only P6.p will receive high amount of LIN-3 (EGF). This surge of ligand during vulval development activates the LET-60/LIN-45/MPK-1 signalling cascade in P6.p, leading to CFP expression exclusively in the P6.p daughter cells (Figure 2A; upper panel). When an attenuator of the pathway is depleted, expression of CFP is activated in VPCs that normally do not express CFP (Figure 2A; lower panel). We call this effect ectopic expression and measure its incidence as the percentage of animals presenting ectopic expression (Figure 2B) and by analysing the frequency of expression in each VPC (Figure 2C and D). We depleted all 28 candidates using RNAi or genetic mutations where available (14 cases) and analysed the percentage of animals presenting ectopic CFP expression. This approach identified eight candidates (Table 2). One candidate, BET-1, was of particular interest, since the incidence of animals presenting ectopic CFP expression is high and its role as an attenuator of LET-60-mediated signalling in the vulva still uncharacterised. Thus, we focused on BET-1, a protein previously shown to recognise acetyl-lysine on histone tails and to maintain the cell fate of various lineages [22]. 

**Table 2:** Percentages of animals presenting ectopic expression of *egl-17::cfp* during vulval development.

allele or RNAi	*egl-17p::cfp* assay	N
*bet-1 (gk425)* ^†^	71%	51
*mys-1 (RNAi)* ^†^	39%	31
*cdt-2 (RNAi)* ^†^	25%	16
*met-2 (n4256)* ^†^	14%	29
*rbbp-5 (RNAi)* ^†^	11%	19
*wdr-5 (ok1417)* ^†^	10%	59
*set-1 (RNAi)* ^†^	7%	28
*jmjd-5 (RNAi)* ^†^	7%	30
*brd-1 (ok1623)*	4%	75
*uba-2 (RNAi)*	4%	27
*chd-3 (ok1651)*	3%	30
*gei-17 (RNAi)*	3%	30
*mes-2 (RNAi)*	3%	31
*cec-5 (RNAi)*	3%	33
*hda-2 (ok1479)*	3%	38
*hpl-2 (ok916)*	3%	38
*wdr-5.2 (ok1444)*	0%	29
*athp-1 (RNAi)*	0%	26
*dpff-1 (RNAi)*	0%	29
*cec-9 (RNAi)*	0%	29
*jmjd1.1 (hc184)*	0%	29
*F47D12.9 (RNAi)*	0%	30
*gei-8 (ok1671)*	0%	15
*hda-6 (ok3311)*	0%	15
*lin-53 (n833)*	0%	33
*mes-4 (RNAi)*	0%	30
*tag-250 (ok1332)*	0%	31
*rcor-1 (ok727)*	0%	38

^†^ indicates candidates showing above background ectopic expression, typically around 5%.

Our initial characterisation of BET-1 as an attenuator of LET-60 signalling (Table 2) was performed using the *bet-1*_*gk425*_ allele. We verified this attenuator activity using an additional allele (*bet-1*_*ok46*_; [22]), and RNAi. Both genetic alleles are predicted to cause strong loss of function since both strains need to be maintained balanced. The *bet-1*_*gk425*_ allele is a small deletion removing 226 and 96 nucleotides respectively up- and down-stream of the start codon. It was suggested that methionine 86, still present in *bet-1*_gk425,_ could be used as an alternative start codon, producing a BET-1 missing half of the first bromodomain [22]. The *bet-1*_*os46*_ allele introduces a nonsense mutation changing tryptophane 62 into a stop codon [22]. The *bet-1*_*os46*_ allele is most likely a null allele. We found that the *bet-1*_*ok46*_ allele produced a similar effect on *egl-17p::cfp* expression to the *bet-1*_*gk425*_ allele (~75% *vs* ~80%; Figure 2B). In addition, we found that reducing BET-1 levels by RNAi treatment gave similar levels of ectopic *egl-17p::cfp* expression as using heterozygous *bet-1*_*gk425*_ animals (~15% *vs* ~20%; Figure 2B), consistent with the knock down effect produced by the RNAi treatment. Thus, using different means to deplete BET-1 consistently resulted in an increase in LET-60-mediated signalling, indicating that BET-1 could be a novel attenuator of the LET-60 pathway acting during vulval development.

How does the effect of depleting BET-1 compares to depleting known attenuators of the LET-60 pathway? To obtain a relative appreciation of BET-1’s ability to attenuate LET-60 signalling, we compared inactivation of *bet-1* to that of known attenuators of the LET-60 pathway, gap-*1* [44] and *smo-1* [9,20,24]. We observed that inactivation of *bet-1* produces a more penetrant effect that the inactivation of gap-*1* (~75% *vs* 35%; Figure 2B). On the other hand, inactivation of *bet-1* appears similar to *smo-1*_*lf*_ (~75% *vs* 80%; Figure 2B), which is also a previously characterised attenuator of the LET-60 pathway [9,20,24]. Thus, BET-1’s ability to attenuate the LET-60 pathway during vulval development is comparable to that of other previously characterised attenuators.

Depletion of BET-1 and SMO-1 gave comparable incidence of animals showing ectopic CFP expression (Figure 2B). Since sumoylation is a post-translational modification, it is plausible that attenuation by the SUMO pathway involves SUMO-modifications of many targets. Thus, SUMO-mediated attenuation of LET-60 signalling should be more potent than than the attenuator activity of one of its target. To address this point, it is necessary to obtain the relative levels of attenuation provided by SMO-1 and BET-1 at a cellular level, i.e. in each VPC descendants. To achieve this, we examined the frequency of ectopic CFP expression for each VPC’s descendants (P3.px-P8.px) (Figure 2C and D). If, as expected SMO-1 were to act on multiple targets to attenuate the LET-60 signalling pathway, we should find that depletion of SMO-1 gave a greater effect on ectopic expression of CFP than BET-1. We found that in *smo-1*_*lf*_ animals, any of the VPCs can show ectopic expression of CFP, whereas in *bet-1*_*lf*_ animals, ectopic expression is rarely observed in P3.p descendants (Figure 2C). Further, the frequency of ectopic expression in descendants of P4,5.p and P7,8.p is generally lower (~50%) than in the case of *smo-1*_*lf*_ animals (Figure 2C). As previously shown [31], all control animals display *egl-17p::cfp* expression specifically in descendants of P6.p, and not in any of the other VPCs (P3,4,5.p and P7,8.p). Thus, it is likely that the sumoylation pathway acts upon additional targets to attenuate LET-60 signalling during vulval development. This is consistent with our screen having identified multiple SUMO interactors.

We next addressed whether the ectopic expression of *egl-17::cfp* observed in absence of BET-1 is dependent upon LET-60, as previously shown for other attenuators [31]. We performed let-*60*(*RNAi*) in wild type animals, and accordingly, we found reduced incidence of *egl-17p::cfp* expression in P6.p descendants (~50%; Figure 2D). We next performed let-*60*(*RNAi*) in the *bet-1*_*lf*_ background and observed over two fold reduction in the incidence of *egl-17p::cfp* ectopic expression in any of the VPCs (Figure 2D). Thus, ectopic *egl-17p::cfp* expression produced by the absence of *bet-1* requires LET-60, providing further evidence that BET-1 is a novel attenuator of the LET-60 signalling pathway.

LET-60 signalling in the VPCs is activated by the LIN-3 (EGF) ligand that emanates from the anchor cell [45]. An increase in LET-60 signalling could therefore be explained by an increase of LIN-3 through duplication of anchor cells [46]. To rule this out, we tested for the presence of duplicated anchor cells in *bet-1*_*lf*_ animals using an anchor cell marker, *zmp-1::yfp*, coupled to *egl-17::cfp* [47,48]. We observed that all *bet-1*_*lf*_ animals that produced ectopic *egl-17::cfp* expression had one anchor cell (62% ectopic *egl-17::cfp*; n=37), ruling out anchor cell duplication as the cause of hyperactivated LET-60 signalling. 

In conclusion, we identified eight candidates acting as attenuators of the LET-60 pathway using the reporter *egl-17p::cfp* reporter assay. Out of these, we found that *bet-1*_*lf*_ displays very strong ectopic *egl-17p::cfp* expression, an indicator of hyperactivated LET-60 signalling. In addition, BET-1 was identified exclusively in the *smo-1*_lf_ heterozygous background (enhancer) group (Table 1 and 2). Genetic interactions observed when both gene products are reduced, but not when both gene products are absent (see below) is consistent with a physical interaction between these gene products, herein BET-1 and SMO-1. 

### Simultaneous depletion of BET-1 and SMO-1 does not elevate LET-60 signalling further than their individual depletion

**Figure 3:**
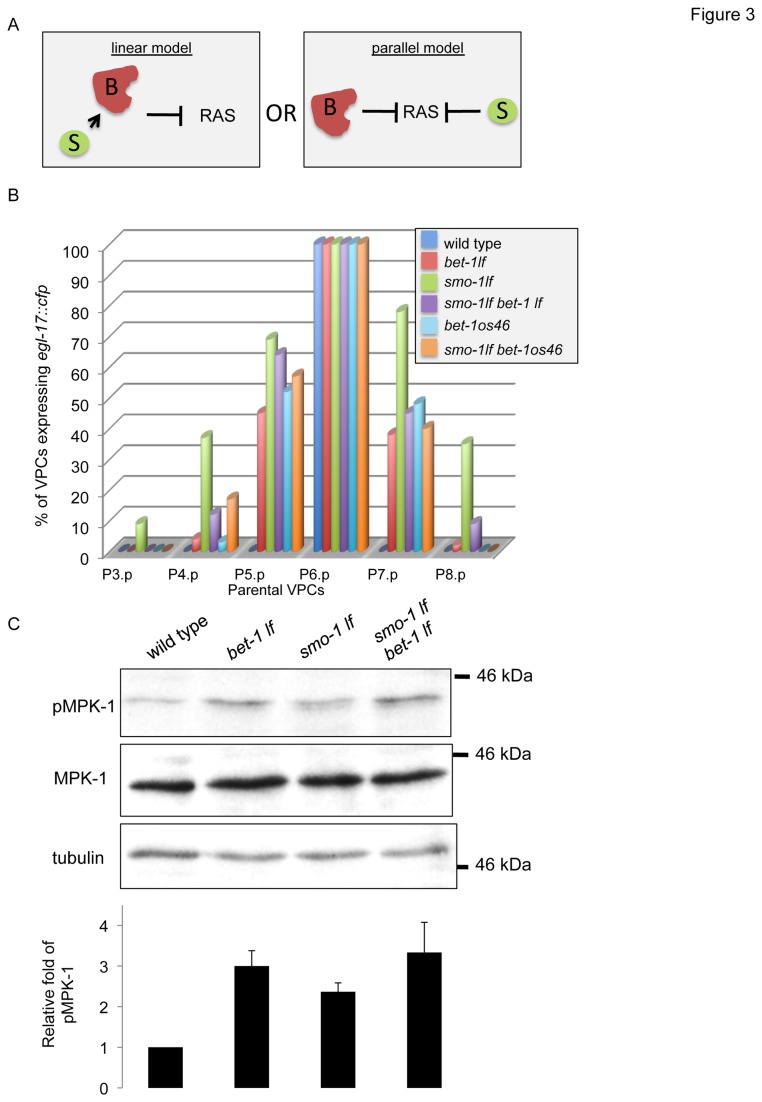
Comparative analysis between the double *smo-1*_*lf*_ bet*-*1_*lf*_ mutant and the respective single mutants. A) Two alternative models (linear and parallel) for attenuation of the LET-60(RAS) signalling pathwayby SMO-1 and BET-1. B) The percentage of VPCs expressing *egl-17::cfp* is indicated on the y axis. Each bar represents the descendants of the indicated parental VPC. The genotype analysed is indicated in the inset legend. We analysed a minimum of 40 animals for each genotype. B) Western blots performed in triplicate, and prepared from larval stage 4 (L4) against phosphorylated MPK-1 (top panel), MPK-1 (middle panel) and tubulin (bottom panel). Quantification of pMPK-1 is relative to tubulin levels.

The possibility that BET-1 could interact physically with SMO-1 prompted us to analyse the effect that the double *smo-1*_*lf*_ *bet-1*_*lf*_ mutant has on LET-60 signalling. If BET-1 needs to be sumoylated (or recognise a sumoylated protein) to attenuate LET-60 signalling, then the absence of both BET-1 and SMO-1 would have the same effect as lacking either of these (Figure 3A; linear model). On the other hand, if BET-1 does not need to be sumoylated (or to recognise a sumoylated protein) to attenuate LET-60 signalling, then the double mutant would have a greater effect on LET-60 signalling than each single mutant (Figure 3A; parallel model). We used two readouts of LET-60 signalling to determine which of these models is correct: *egl-17p::cfp* expression and levels of phospho-MPK-1 (p-MPK-1). MPK-1 becomes phosphorylated following activation of the LET-60 signalling cascade and is a quantitative readout of LET-60 signalling activity [49]. Using the *egl-17p::cfp* assay, we found that the double *smo-1*_*lf*_ *bet-1*_*lf*_ mutant or the double *smo-1*_*lf*_ *bet-1*_*os46*_ does not increase ectopic expression of *egl-17::cfp* beyond what is already observed in single mutants (Figure 3B). Similarly, using Western blots to assess the levels of p-MPK-1, we found that the double *smo-1*_*lf*_ *bet-1*_*lf*_ mutant does not increase p-MPK-1 levels beyond the levels already observed in single mutants; *bet-1*_*lf*_ and *smo-1*_*lf*_ display ~3 and ~2.5 fold increases, respectively, and the double *smo-1*_*lf*_ *bet-1*_*lf*_ a ~3 fold increase (Figure 3C). The increases in p-MPK-1 levels observed in single mutants are consistent with their role in attenuation of LET-60 signalling. Thus, the results from the *egl-17p::cfp* assay and the Western blots against p-MPK-1 are consistent with the linear model and suggest that BET-1 could physically interact with SMO-1.

### BET-1 interacts with SUMO and can be sumoylated *in vitro*

**Figure 4:**
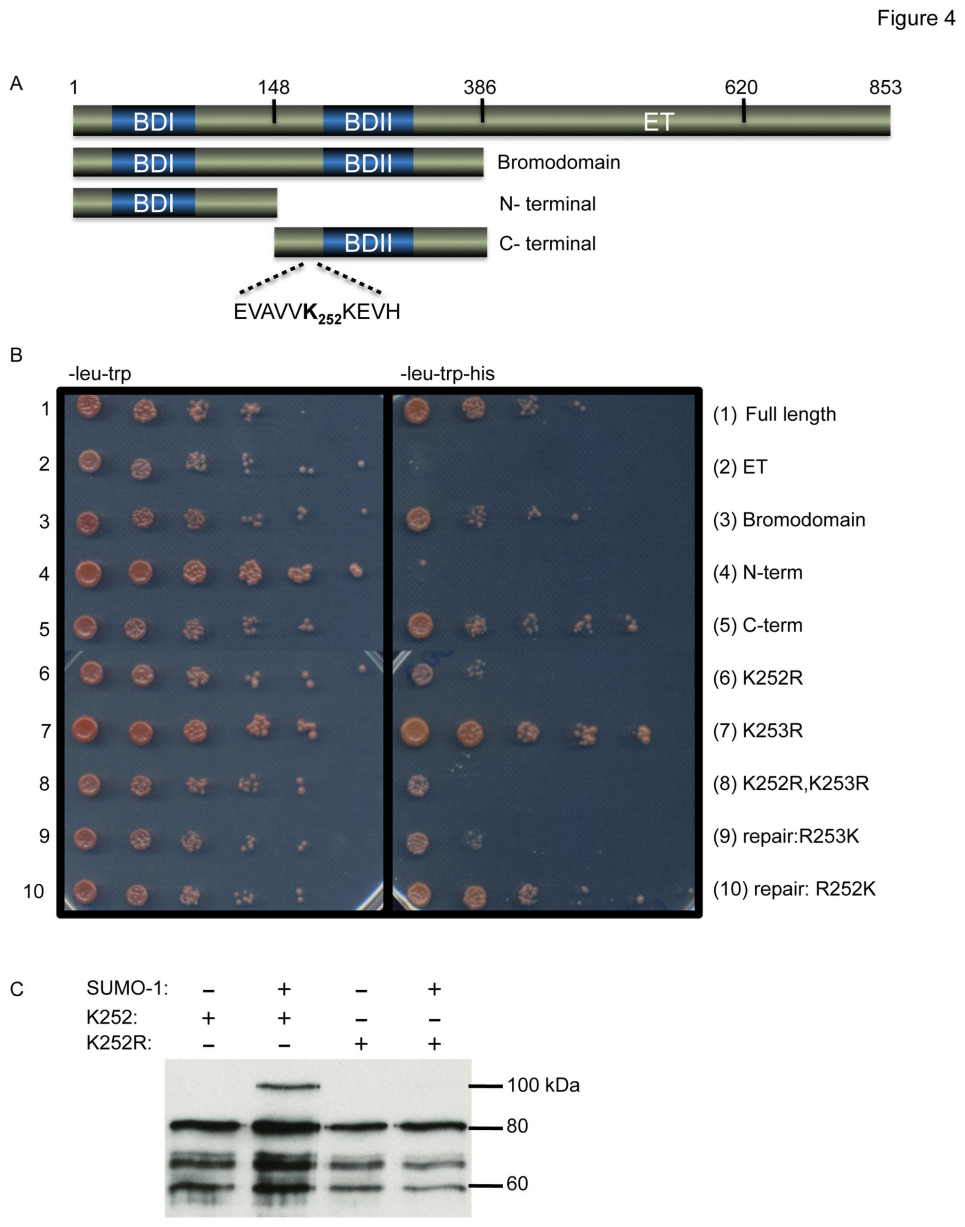
BET-1 can be sumoylated *in* *vitro*. A) A schematic map depicting BET-1 full length and the different fragments used in yeast two-hybrid and for the *in* *vitro* sumoylation assay. B) The yeast two-hybrid assay shows that BET-1 and SMO-1 interact. The interaction requires lysine 252 within the C-terminal part of the bromodomain. The fragment from amino acid 614 to 853 auto activates and could not be tested for interactions. The photographs show the results from six serial (1 in 5) dilutions to allow semi-quantitative comparisons between conditions. Omitted amino acids are indicated on top left hand side. C) The *in* *vitro* sumoylation assay analysed by Western blot against MBP shows that lysine 252 is sumoylated. The expected size of MBP fused to the bromodomain is roughly 80kDa and its sumoylated form corresponding to an added SUMO-1, branching out of the bromodomain, produces a shift of ~20kDa. The bands detected below are likely to be degradation products and/or incomplete translation products.

To provide evidence that SMO-1 and BET-1 physically interact, we used the yeast two-hybrid system, and an *in vitro* sumoylation assay. Similarly to other studies using the yeast two-hybrid system [30,50,51], we used SMO-1 or UBC-9 as prey and BET-1 full length and a number of fragments as baits (Figure 4A). We found that BET-1 full length as well as the fragment containing the bromodomains can interact with SMO-1 or UBC-9, the E2 enzyme necessary for sumoylation. In contrast, a fragment containing the extra terminal domain did not interact with either SMO-1 or UBC-9 (Figure 4B and Figure S1). We further dissected the bromodomain-containing fragment and found that a peptide covering the second bromodomain is sufficient for the interaction (Figure 4B). This peptide contains a consensus SUMO site (Ψ-K-x-D/E) at lysine 252. We tested whether lysine 252 is involved in the interaction. We mutated lysine 252 and an additional lysine, lysine 253, into arginines; arginine is also a positive and hydrophilic residue. We found that the interaction with SMO-1 or UBC-9 is lost if lysine 252 is mutated. In contrast, there is no effect on the interaction when lysine 253 is mutated (Figure 4B and Figure S1). Mutating both together gave the same result as mutating lysine 252 on its own (Figure 4B). We also changed each mutated residue back into lysine and recovered the interaction with SMO-1 when arginine 252 was changed back into a lysine (Figure 4B). Reinstating lysine 253 had no effect (Figure 4B). We tested six additional lysines (264, 276, 277, 304, 313, and 315) within the second bromodomain, but none of these were required for the interaction (Figure S1). Thus, these experiments indicate that BET-1 can physically interact with SMO-1 and UBC-9 and that lysine 252, within a sumoylation consensus site, is required for that interaction to occur. 

We next tested whether BET-1 can be sumoylated *in vitro* using a sumoylation assay. We used MBP-tagged proteins purified from *E. coli* to test either the wild type bromodomain or the mutated form that can no longer interact with SMO-1 or UBC-9 in yeast two-hybrid. The transfer of the SUMO peptide to a targeted lysine will add about 12kDa onto the theoretical mass of the substrate, but the branching structure often produces shifts of around 20kDa. We used Western blotting against MBP to detect an effect on migration. We found a slow migrating band only when the wild type form of the bromodomain was used in the presence of SUMO-1 (Figure 4C). In contrast, when lysine 252 is mutated into an arginine, we could not detect this slow migrating band (Figure 4C). This shows that the bromodomain of BET-1 can be sumoylated *in vitro* and that lysine 252 is essential to the process. 

## Discussion

The main goal of our study was to uncover chromatin regulators genetically interacting with the sumoylation pathway to prevent hyperactivation of the conserved RAS/RAF/MAPK signalling pathway with a particular interest on candidates that could potentially be sumoylated. There is strong evidence that SUMO-modified transcription factors can recruit chromatin regulators to attenuate the LET-60(RAS) pathway in *C. elegans* [20]. However, it was unclear whether sumoylation of chromatin regulators was required to attenuate LET-60 signalling. Here, we provide multiple lines of evidence that a sumoylated BET-1 influences attenuation of LET-60 signalling: loss of both BET-1 and SMO-1 together produces the same effect on LET-60 signalling as the loss of either of them; BET-1 physically interacts with SMO-1 and UBC-9; and BET-1 can be sumoylated *in vitro*. Since BET-1 is a reader of the epigenetic code, specifically of acetyl-lysines on histone tails, it is interesting to speculate that sumoylation of BET-1 could regulate its ability to bind modified acetylated histone tails. 

Acetylation of lysines on histone tails is strongly associated with activation of transcription. Acetyl groups neutralise the positive charges found on histones, thereby weakening the interaction with the negatively charged phosphate groups on DNA. This loosens the chromatin structure and facilitates transcription factors’ access to the underlying DNA. In addition, acetyl-lysines can serve as docking sites for double bromodomain-containing proteins, which leads to recruitment of complexes that can positively regulate transcription [52,53]. We envision two possible and non-mutually exclusive mechanisms by which BET-1 could, by positively regulating transcription, impact on attenuation of LET-60 signalling. Firstly, BET-1 could be required to activate the expression of genes that attenuate LET-60 signalling, perhaps MAPK phosphatases, and thus aid in establishing a negative feedback loop. Alternatively, BET-1 could influence the threshold of a RAS/RAF/MAPK ‘competence zone’ [54]. Thus, in absence of BET-1, the threshold of this competence zone would be lowered and the activation of the RAS/RAF/MAPK signalling pathway thereby facilitated. This mechanism implies that BET-1 would regulate the expression of genes responsible to elevate the threshold of the competence zone. Intriguingly, both mechanisms infer that BET-1 would act on a relatively limited number of specific enhancers/promoters, an activity not normally attributed to chromatin regulators. However, recent work has shown that BRD4 (a human homolog of BET-1) displays specific activity towards MYC by acting in a concentration dependent manner on super-enhancers [55]. It is thus possible that BET-1 could also act at super-enhancers in *C. elegans*, but future work will be required to address this point.

Our data strongly suggest that BET-1 can be SUMO-modified, leading us to speculate that sumoylation may regulate BET-1’s ability to associate with acetyl-lysines on histone tails. This could occur *via* three mechanisms: i) Stabilisation of BET-1 expression levels; ii) increase in BET-1’s affinity to acetylated histones; or iii) increase in BET-1’s capacity to access acetylated histones. i) As a small ubiquitine like modifier, SUMO can compete with ubiquitination and can protect against degradation [56]. ii) Addition of a SUMO peptide to a protein can cause conformational changes enhancing affinity for its target [57]. Finally, iii) sumoylation can increase protein solubility [58]; in the case of BET-1 an increased in solubility may facilitate access to higher order chromatin or allow dynamic changes in its genomic location. 

In addition, SUMO-modified BET-1 could be recognised by proteins containing a SUMO interaction motif (SIM) and as such could recruit additional chromatin regulators. Such function has been demonstrated for the human transcriptional repressor KAP1. KAP1 contains a bromodomain which, when sumoylated, recruits the histone methyltransferase SETDB1 and the NuRD complex component CHD3 to gene promoters, hence leading to repression of transcription [41]. Interestingly, our screen has identified the *C. elegans* SETDB1 homolog (MET-2) as well as CHD-3, raising the possibility that MET-2 and CHD-3 could associate with SUMO-modified BET-1. On the other hand, SUMO-modified BET-1 could also be recognised by other SIM-containing proteins involved in activation of transcription such as the NuA4/TIP60 histone acetyltransferase complex [22]. Since attenuation of LET-60 signalling by BET-1 is likely to occur through establishment and/or maintenance of a transcriptional state, we speculate that sumoylation of BET-1 provides a mechanism that regulate this transcriptional state. 

Finally, most RNAi screens performed in sensitised genetic backgrounds inform on only one type of genetic interaction. Depending on the screen design, screens will identify either enhancers (if performed in a reduced function background) or synthetic interactions (if performed in a loss of function allele). Here, using a *smo-1*_*lf*_ GFP balanced strain, we performed one screen that has identified both enhancers and synthetic interactors. Subsequently, these candidates can then be grouped into enhancers and/or synthetic classes and the information used to help deciding which candidates should be studied in details. This approach was useful to identify BET-1 as a novel attenuator of the LET-60(RAS) signalling regulated by the sumoylation pathway, possibly by direct SUMO modification within the second bromodomain. 

## Supporting Information

Figure S1 **Yeast two-hybrid showing that SMO-1 and UBC-9 can interact similarly with the C-terminal domain of BET-1.** K (lysine) mutated in R (arginine) at the indicated amino acid.
(TIF)

Table S1 **The list of genes targeted with their associated domains and the library source of the RNAi clones.**
(XLSX)

Table S2 **The PFAM identification number used to identify the chromatin regulator set.**
(XLSX)

Table S3 **The list of all strains used with their complete genotype.**
(XLSX)
