# Supplementary figures and images for: An RNAi-Based Dimorphic Genetic Screen Identified the Double Bromodomain Protein BET-1 as a Sumo-Dependent Attenuator of RAS-Mediated Signalling
Source: PLoS One. 2013 Dec 10;8(12):e83659. doi: 10.1371/journal.pone.0083659 (PMC3862036; doi:10.1371/journal.pone.0083659)

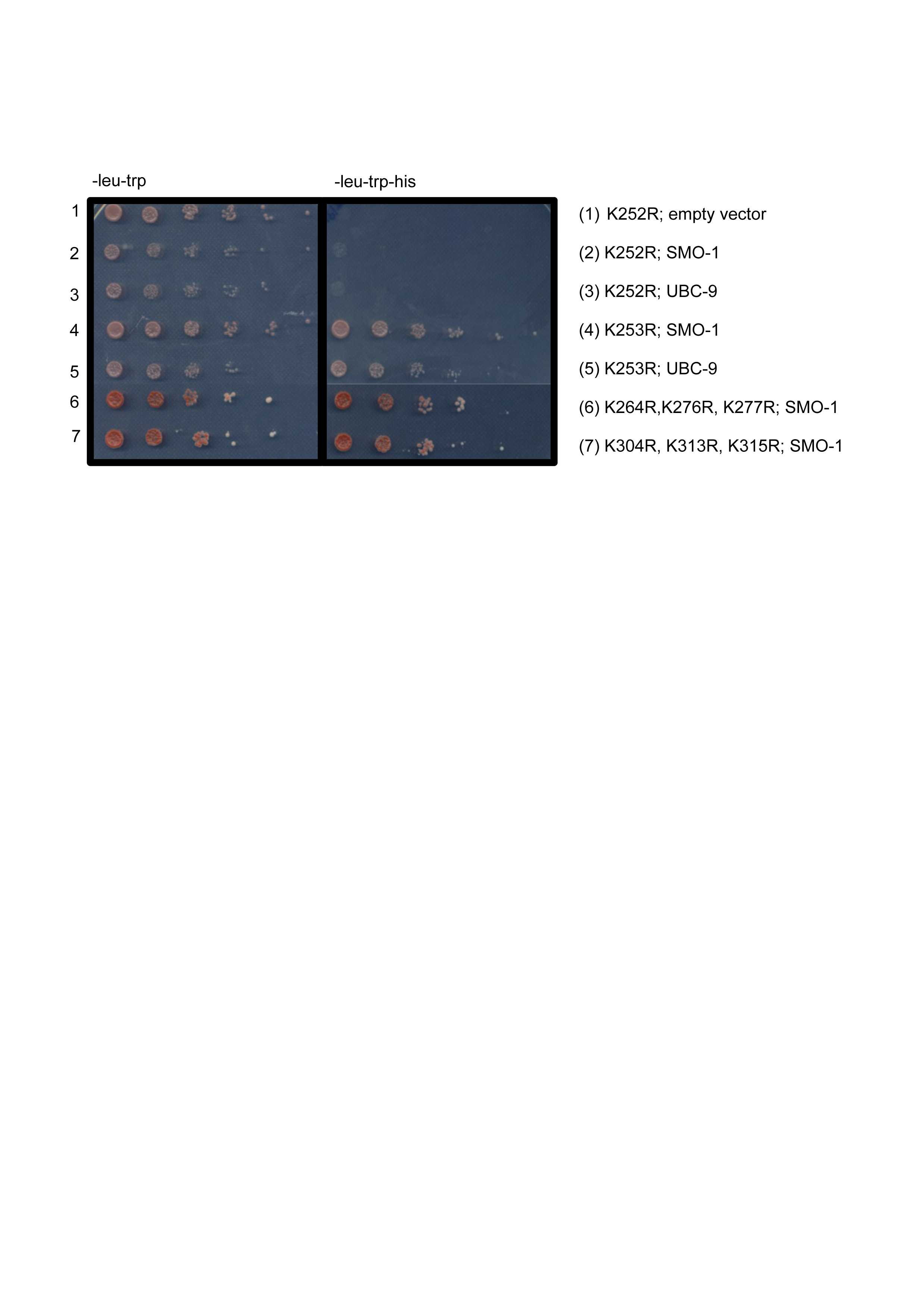

Supplement: Figure S1 — Yeast two-hybrid showing that SMO-1 and UBC-9 can interact similarly with the C-terminal domain of BET-1. K (lysine) mutated in R (arginine) at the indicated amino acid. (TIF) [file pone.0083659.s001.tif]
